# IL-6/STAT3 Is a Promising Therapeutic Target for Hepatocellular Carcinoma

**DOI:** 10.3389/fonc.2021.760971

**Published:** 2021-12-15

**Authors:** Junnv Xu, Haifeng Lin, Gang Wu, Mingyue Zhu, Mengsen Li

**Affiliations:** ^1^ Hainan Provincial Key Laboratory of Carcinogenesis and Intervention, Hainan Medical College, Haikou, China; ^2^ Department of Medical Oncology, Second Affiliated Hospital, Hainan Medical College, Haikou, China; ^3^ Institution of Tumour, Hainan Medical College, Haikou, China

**Keywords:** IL-6/STAT3 signal, hepatocellular carcinoma, targeted treatment, IL-6 receptor, malignant transformation

## Abstract

Hepatocellular carcinoma (HCC) is a common malignant tumor of which the occurrence and development, the tumorigenicity of HCC is involving in multistep and multifactor interactions. Interleukin-6 (IL-6), a multifunctional inflammatory cytokine, has increased expression in HCC patients and is closely related to the occurrence of HCC and prognosis. IL-6 plays a role by binding to the IL-6 receptor (IL-6R) and then triggering the Janus kinase (JAK) associated with the receptor, stimulating phosphorylation and activating signal transducer and activator of transcription 3 (STAT3) to initiate downstream signals, participating in the processes of anti-apoptosis, angiogenesis, proliferation, invasion, metastasis, and drug resistance of cancer cells. IL-6/STAT3 signal axes elicit an immunosuppressive in tumor microenvironment, it is important to therapy HCC by blocking the IL-6/STAT3 signaling pathway. Recent, some inhibitors of IL-6/STAT3 have been development, such as S31-201 or IL-6 neutralizing monoclonal antibody (IL-6 mAb), Madindoline A (Inhibits the dimerization of IL-6/IL-6R/gpl30 trimeric complexes), C188-9 and Curcumin (Inhibits STAT3 phosphorylation), etc. for treatment of cancers. Overall, consideration of the IL-6/STAT3 signaling pathway, and its role in the carcinogenesis and progression of HCC will contribute to the development of potential drugs for targeting treatment of liver cancer.

## Introduction

Cellular signaling pathways refer to the process of intracellular biochemical effects after extracellular signals act on membranal or intracellular receptors. The interleukin-6 (IL-6)/signal transducer and activator of transcription 3 (STAT3) signaling pathway participates in various physiological processes, including cell growth, differentiation, and immune regulation. Many studies have shown that abnormal IL-6/STAT3 signaling pathways play a crucial role in tumorigenesis and development. Continuous activation of the IL-6/STAT3 signaling pathway has been detected in liver cancer, lung cancer, breast cancer, ovarian cancer, gastric cancer and other cancers (1–5), and IL-6/STAT3 may be a promising biotarget to prevent and treat cancer.

Hepatocellular carcinoma (HCC) is a serious worldwide disease, with over 900,000 new HCC cases and 830,000 deaths in 2020 (6). China is a populous country, and the number of new tumor cases and the number of deaths are the highest in the world. In 2020, 410,000 new HCC cases ranked fifth and 390,000 HCC deaths ranked second worldwide (6, 7). The etiology and exact molecular mechanism of HCC genesis are not fully clear, and its etiology is currently considered to be a multifactor, multistep complex process. Hepatitis B virus (HBV) and hepatitis C virus (HCV) infection are the primary causes, during infected with HBV or HCV in liver tissue, inflammation is induced by the hepatitis viruses, the inflammatory cells secreted IL-6 to activate STAT3 signal pathway to stimulate tumorigenicity (8–10). Although great progress in treatment of HCC(including surgery, targeted therapy and immunotherapy), the treatment effect is still not satisfactory (11). Therefore, it is important to explore the occurrence and development mechanism of HCC, and seek a potential novel biotarget for treatment of HCC.

The occurrence and development of HCC is associated with disorders of many signaling pathways. IL-6/STAT3 is one of the key signaling pathways involved in HCC occurrence and plays an important role in the initiation, development, invasion and metastasis of HCC cells (12). IL-6 family cytokines are commonly used of the signal-transducing receptor chain glycoprotein 130 (gp130) to transduce the growth signal in cells, these cytokines play a crucial role in promoting carcinogenesis and progression of HCC (13). Accumulating evidences indicated the pro-inflammatory, IL-6 in tumor microenvironment has a trait to activate IL-6/STAT3 signal pathway, and promote the development of cancer, include HCC and the aggressiveness of HCC cells (14–16), IL-6 is highly expressed in liver cancer tissue and loaded in serum, and overexpressed IL-6 is closely associated with the staging, severity, and prognosis of HCC (17). IL-6, as an inflammatory-related tumor cytokine, activates a series of factors downstream by activating the IL-6/STAT3 signaling pathway, leading to the occurrence of malignant behaviors, such as HCC cell proliferation, drug resistance, invasion and metastasis (18, 19).

Therefore, we review the role of the IL-6/STAT3 signaling pathway in HCC occurrence and development, and describe the current therapeutic strategies for targeting treatment of HCC in the IL-6/STAT3 signaling pathway. In recent years, the role of the IL-6/STAT3 signaling pathway in the tumorigenicity and development of HCC has become increasingly valued. Blocking this signaling pathway may inhibit the development of liver cancer, and many drugs with molecular targets have been used in the clinical diagnosis and treatment of cancers.

## The IL-6/STAT3 Signaling Pathway

The role of IL-6/STAT3 signaling pathway in stimulating origination of inflammation and cancer was initially discovered by researchers, it was found that interferons (IFNs) and IL-6 were able to regulate the activity of downstream signaling molecules, which play an important role in tumorigenesis and development by regulating downstream transcription factors and can serve as a potential target for cancer therapy.

### Constituents of the IL-6/STAT3 Signaling Pathway

**Figure 1:**
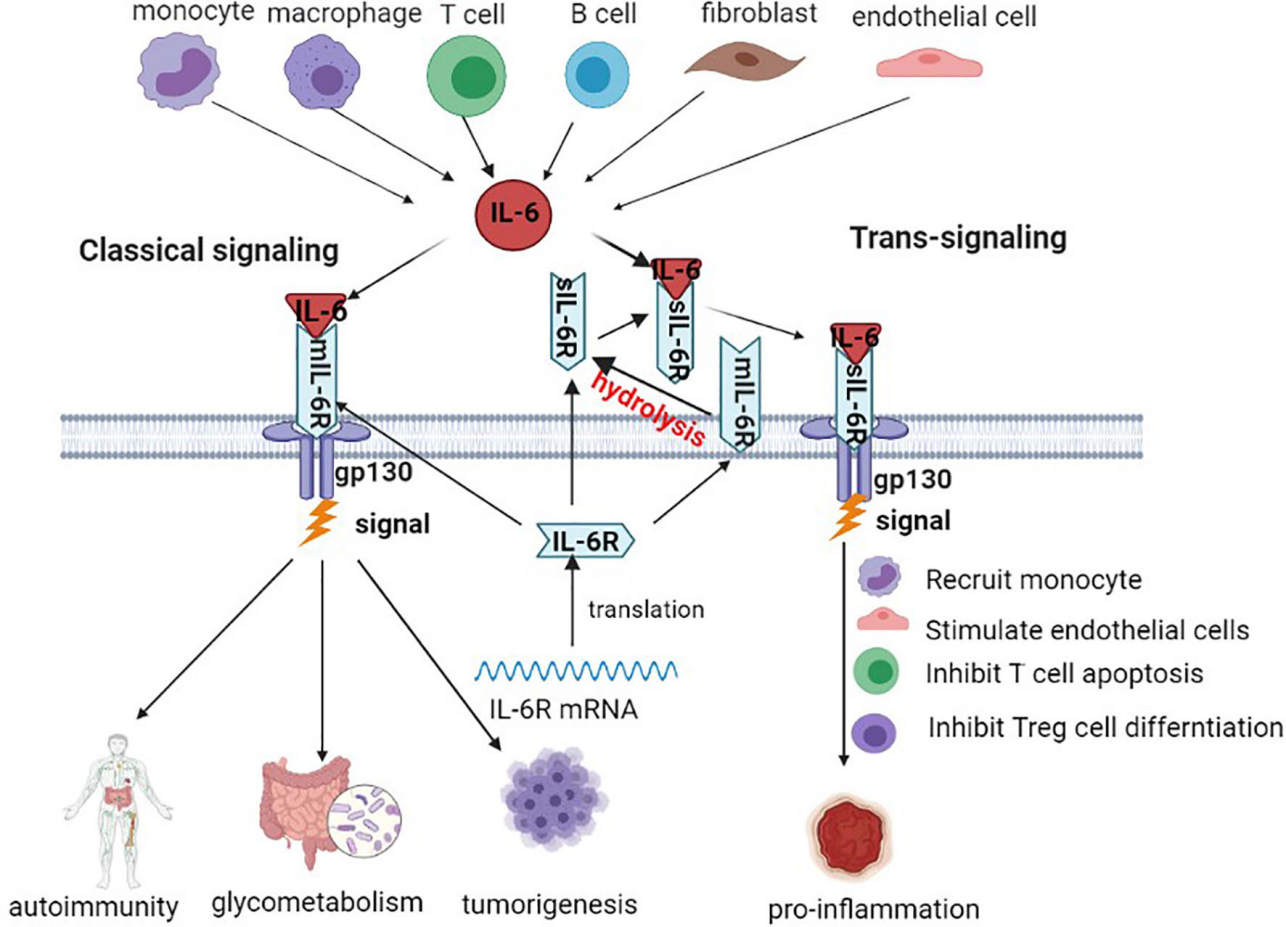
Production source and main signaling pathway of IL-6. IL-6 transduction is mainly produced by monocytes, macrophages, T cells, B cells, fibroblasts, etc. IL-6R binds to the surface of the cellular membrane through the classical pathway. In the signaling transduction pathway, IL-6 binds to sIL-6R and then initiates signaling transduction.

IL-6 is a multifunctional inflammatory cytokine, a small molecular polypeptide consisting of four α helices with a molecular weight of 19-228 kD, with 184 amino acid residues located in the p21 region of chromosome 7 (20). Studies have shown that bone marrow stem cells secrete IL-6, and tumor cells themselves and tumor-associated macrophages (TAMs) also release IL-6. Meanwhile, IL-6 can be subjected to upregulation of interleukin-1β (IL-1β), tumor necrosis factor-α (TNF-α), and stress reactions. The expression of IL-6 is very low in normal human cells, with increased serum concentration in patients with hepatitis and liver cancer (21, 22). The most fundamental action of IL-6 and plays multipotent functions due to bind with its receptor. The IL-6 receptor(IL-6R) system is mainly composed of the IL-6 ligand binding chain and signal transduction chain, namely, IL-6R and gp130. IL-6R is usually found in many cells, such as hepatocytes, monocytes, macrophages and neutrophils, and is generally divided into membrane-bound IL-6R (mIL-6R) and soluble IL-6R (sIL-6R). mIL-6R is located on the cellular membrane surface, and sIL-6R is formed by protein hydrolysis of mIL-6R on the cellular membrane or directly by splicing mRNA during the translation phase (23). In the classical signaling transduction pathway, IL-6 contacts mIL-6R on the membrane, causes dimerization and then starts transduction of signaling, mainly participating in autoimmunity, metabolism, tumor development, etc. During signal transduction, first, IL-6 binds with sIL-6R, and then the complex binds with membrane gp130. This binding pattern plays a main role in inducing inflammatory reactions (24–26) (see **Figure 1**).

**Figure 2:**
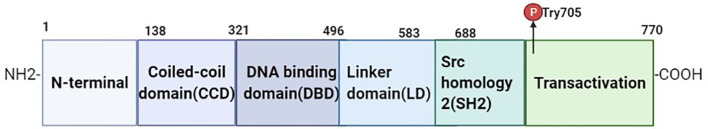
Structure of STAT3 protein. STAT3 consists of six major components, including CCD, DBD, LD, SH2, N-terminal, and transactivation. The N-terminal domain mediates the interaction between STAT3, promoter binding and transcription mechanisms. CCD domains promote the interaction of regulatory proteins and transcription factors. The DBD is involved in the regulation of STAT3 gene promoters. The SH2 domain forms a dimer by binding the phosphorylation of the STAT3 monomer with the Tyr705 region site, which is responsible for transcriptional activation of the target gene.

Signal transducers and activators of transcription proteins (STATs) are important in cellular signaling and include seven families: STAT1, STAT2, STAT3, STAT4, STAT5A, STAT5B and STAT6 (27). STAT3 was originally found by Shi et al (28) as an acute phase reaction factor (APRF) in IL-6 signaling when studying interferon-induced gene transcription in 1996. STAT3 is a family of cytoplasmic proteins, and its encoding gene is located on chromosome 12, consisting of 750-795 amino acid residues with a molecular weight of 89-99 kDa. Its activation sites are primarily the SH2 domain (Src homology 2 domain) as well as tyrosine phosphorylation site 705 (Tyr705) and serine phosphorylation site 727 (Ser727) in the transcriptional activation region. The core structure of STAT3 mainly consists of a coiled-coil domain (CCD), DNA binding domain (DBD), linker domain (LD), Src homology 2 (SH2), amino acid terminal region, and carboxy-end trans activation region (29), where Tyr705, SH2 and DBD play a key role in STAT3 functions (30). The DBD is structurally an immunoglobulin folding domain that binds to DNA in the form of a dimer and is involved in the transfer of STAT3 from the cytoplasm to the nucleus. The SH2 region is the most conserved domain of the STAT3 protein and is mainly involved in the phosphorylation of tyrosine residues, promoting protein interactions with tyrosine phosphorylation proteins (31). This region has three binding pockets, the phosphorylated Tyr705 (pTyr705) binding site, a side pocket, and a hydrophobic binding pocket, where STAT3 participates in phosphorylation and plays an important role in the phosphorylation of STAT3. During STAT3 activation, the tyrosine and serine residues are phosphorylated by the upstream kinase and identified by the SH2 domain (32) (see **Figure 2**).

### Activation of the IL-6/STAT3 Signaling Pathway

**Figure 3:**
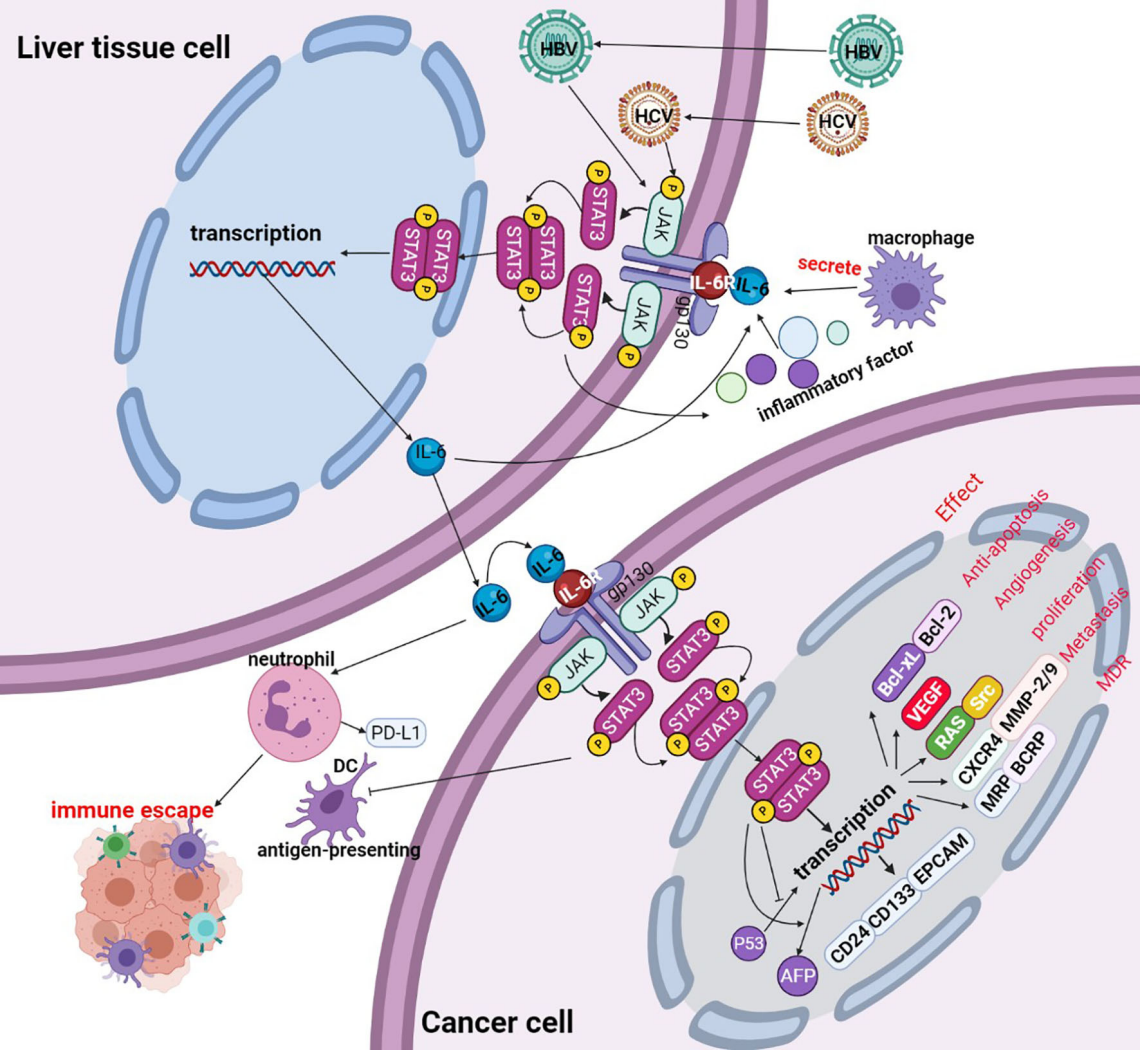
Activation of the IL-6/STAT3 signaling pathway. Liver tissue cell is infected with hepatitis B virus(HBV) or hepatitis B virus(HCV), the viruses promote liver tissue cell to secretion of IL-6, IL-6 binds to the cellular surface receptor, thereby phosphorylating the JAK protein and phosphorylating the STAT3 monomer to form a STAT3 dimer. Phosphorylated STAT3 dimers are transported to the nucleus, promoting the transcription of targeted genes. The activated STAT3 complex is transferred from the cytoplasm to the nucleus to initiate transcription of STAT3 targeted genes (including Bcl-xL, Bcl-2, VEGF, Src, CXCR4, MMP2/9, etc.) and thus participate in cancer cell proliferation, apoptosis, invasion, metastasis etc. PIAS, SOCS, and PTP are negative regulators of IL-6/STAT 3 by inhibiting the activation of JAK or STAT3 phosphorylation itself.

IL-6, as a classic extracellular stimulation factor of this signaling pathway, during the hepatocarcinogenesis and HBV or HCV stimulates initiation of HCC, the secretion of IL-6 is emerged in the microenvironment of liver tissue cells, then IL-6 interaction with its receptor, conformation of IL-6 changes after binding with its receptor, and then activation of gp130 on the cell membrane surface to trigger isodimer formation of gp130, thus leading to activation of Janus kinases(JAKs). After the activation process of JAKs, the binding sites interacting with STAT3 in the cytoplasm are exposed, wherein STAT3 acts primarily with the binding site of the corresponding tyrosine receptor through its SH2 domain. STAT3 binding to the tyrosine binding site to trigger phosphorylation of C-terminal domain tyrosine residues(Tyr705) on STAT3, and simultaneous activation followed by substantial aggregation and activation of STAT3 within the cytoplasm. Phosphorylated STAT3 forms homologous dimers through their SH2 domains, and the dimers are transported from the cytoplasm into the nucleus with the participation of the DBD. Then, the dimers bind to the promoter region of the downstream effector targeted genes, lead to change in the transcript activity of numerous genes, including antiapoptotic genes, angiogenic genes, proliferating genes, transformational genes, and the immune response factors. The expression of apoptosis-, growth- and metastasis-related proteins, such as Bcl-xL, Bcl-2, VEGF, Src, CXCR4, and MMP2/9 were also regulated by phosphorylated STAT3 forms, thus promoting the growth, development and inhibition apoptosis of cancer cells (33) (see **Figure 3**).

### Negative Regulation of the IL-6/STAT3 Signaling Pathway

**Figure 4:**
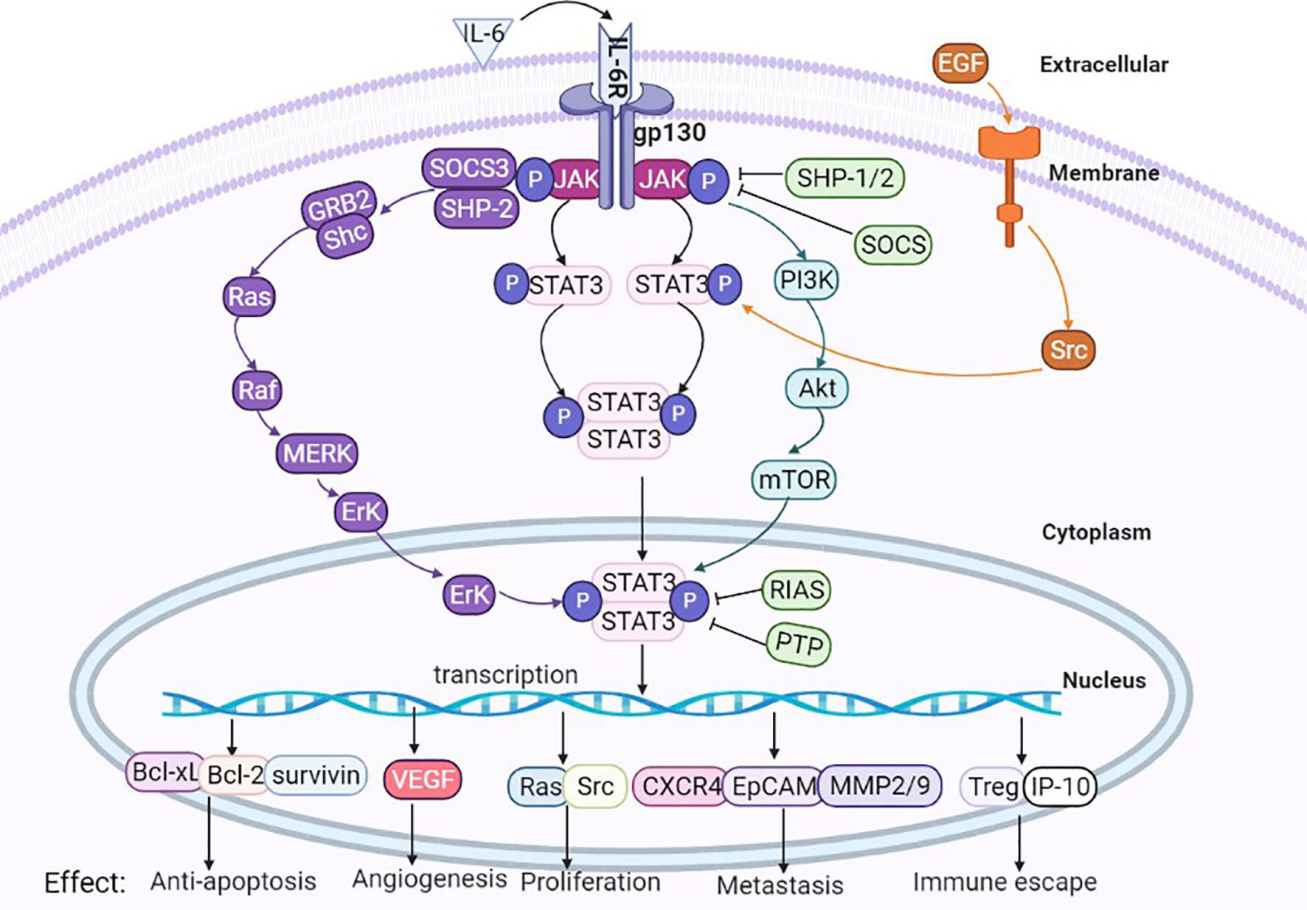
Interaction of IL-6/STAT3 signaling pathways and other pathways. IL-6/STAT3 signaling pathways cross-talk with other signaling pathways, such as Ras, RTK, TNF and PI3K/Akt, and the biological effect of cytokine production is the interaction between many signaling pathways.

A positive/negative feedback pathway exists in cellular signaling. Negative regulation of IL-6/STAT3 mainly includes three classes of negatively regulated proteins: suppressors of cytokine signaling (SOCS), protein inhibitors of activated STAT (PIAS) and protein tyrosine phosphatases (PTPs) (see **Figure 4**).

The SOCS family consists of SOCS1-SOCS7 and CIS, with an N-terminal region, SH2 region and C-terminal SOCS box region. The SH2 region contains an SH2 domain, which can cooperate with N regions to make different SOCS proteins identify different targets by binding to specific cytokine receptors and then regulate various cytokine signal transduction pathways. SOCS molecules are negative feedback regulatory proteins of the classical IL-6/STAT3 signaling pathway, and they inhibit the phosphorylation of STAT3 and the formation of dimers or directly inhibit the phosphorylation of JAK, thus negatively regulating the IL-6/STAT3 pathway to inhibit the continuous proliferation and differentiation of cancer cells (34, 35). Its inhibitory effect on the IL-6/STAT3 pathway also disappears after SOCS inactivation, resulting in continuous proliferation and invasion of cancer cells. Overexpression of the SOCS proteins can inhibit the activity of STAT3 and thus promote apoptosis of cancer cells. Increasing evidence has shown that SOCS is closely related to the initiation and development of HCC (36), and evidence has shown that the absence of the SOCS protein or knockout of the SOCS3 gene in mice, leads to the disappearance of the negative regulation of IL-6/STAT3 by SOCS, thus causing continuous activation of STAT3 and ultimately promoting the occurrence and development of liver cancer (37).

The PIAS family includes PIAS1, PIAS2 (PIASx), PIAS3 and PIAS4 (PIASy), where PIAS3 is the primary specific suppressor of STAT3 and is naturally present in the cytoplasm. On the one hand, PIAS3 specifically binds to STAT3 dimers, thus concealing the DBD of STAT3, and on the other hand, it can bind with the STAT3 monomer to hinder its dimerization (38, 39). A PIAS3-deta peptide can significantly downregulate the expression of the tumor proliferation-related proteins STAT3, pSTAT3, Bcl-2, Cyclin D1, PCNA and c-myc and effectively inhibit the proliferation of HCC cells (40).

The PTP family includes SHP1, SHP2, CD45PTP1B, T-cell PTP (TC-PTP), PTPRT, and PTPBL, where SHP-1 and SHP-2 are the most representative and can bind to a phosphorylated receptor or JAK to dephosphorylate activated molecules, thus blocking the activation of the IL-6/STAT3 signaling pathway. Bard-Chapeau et al. (41) performed selective silencing of SHP-2 in HCC cells, which significantly increased phosphorylation of STAT3 induced by IL-6, thus promoting the transduction of the IL-6/STAT3 signaling pathway. Additionally, SHP-1 plays a strong inhibitory role in HCC epithelial-mesenchymal transition (EMT) by directly lowering pSTAT3 (Tyr705) by exerting its tyrosine phosphatase activity (42).

### IL-6/STAT3 Interacts With Other Signaling Pathways

Classical IL-6/STAT3 signaling pathways are theoretically simple, but they can also interact with other signaling pathways, these signals interlace interaction to exert complex biological effects. (1) Ras signaling pathway: Activated JAKs can phosphorylate tyrosine residues of their associated receptors, leading to the assembly of sites for proteins containing SH2 domains from other pathways, such as SHP-2, which can recruit a large number of GRB2 molecules, and then continuous activation of cascade events, such as the Ras, Raf, MEK, and ERK signaling pathways (43). The IL-6/STAT3 pathway can also indirectly activate the Ras pathway through SOCS3 (44). (2) RTK signaling pathway: Numerous studies verified that STAT3 can be activated through receptor tyrosine kinases (RTKs) (45), and activation of some RTKs, such as epidermal growth factor receptor (EGFR), can cause STAT3 tyrosine phosphorylation through Src kinase. Activation of the RTK pathway results in the upregulation of mitogen-activated protein kinase (MAPK) activity, and MAPK specifically phosphorylates one serine (Ser) in the C-terminus of most STAT3 proteins. Phosphorylation of Ser greatly enhances the transcriptional activity of STAT3. (3) Tumor necrosis factor (TNF) signaling pathway: the TNF signaling pathway interacts with JAK/STAT3 at multiple levels (46). (4) PI3K/Akt signaling pathway: IL-6 binding with its receptor causes phosphorylation of JAK, thus recruiting phosphatidylinositol 3-kinase (PI3K) to the plasmalemma, and a large accumulation of PI3K produces pleckstrin homology (PH) domain binding of Akt. The phosphorylation at Thr308 and Ser473 sites of Akt molecule, leading to changes in the downstream substrate mTOR and thus playing a crucial role in promoting growth, proliferation, survival, differentiation, invasion and metastasis of cancer cells (47). Therefore, the biological effects of IL-6 family cytokines may be involved in the interactions between many signaling pathways (see **Figure 4**).

## The Role of the IL-6/STAT3 Signaling Pathway in the Initiation of Liver Cancer

### Effect of the Il-6/Stat3 Signaling Pathway on the Malignant Transformation of Hepatocytes

#### Effects on Production of Liver Cancer Stem Cells

The existence of cancer stem cells (CSCs) was first proposed by Mackillop in 1983 (48), who argued that tumors were initiated and maintained by a small fraction of CSCs or tumor-initiating cells capable of self-updating and differentiation into different cell lineages. CSCs can be distinguished by various biomarkers, such as CD133, CD24, EpCAM and CD44, and are often considered to cause tumor initiation, development, metastasis, and recurrence. Previous studies have also confirmed the presence of liver cancer stem cells (LCSCs), and many signaling pathways are associated with the maintenance and propagation of LCSCs (49). The IL-6/STAT3 signaling pathway has attracted attention in LCSCs (50), and researchers have reported that the inhibition of LCSCs can be achieved by suppressing the transduction of IL-6/STAT3 signaling (51). Continuously elevated activity of pSTAT3 can increase the expression of the surface marker molecules CD133, EpCAM and CD44 in LCSCs (52). Wan et al. (53) also found that tumor-associated macrophages(TAMs) stimulated STAT3 to promote production of LCSC through the secretion of IL-6; the activation of IL-6/STAT3 signals promotes liver cancer cells to produce LCSC, facilitating the resistance of liver cancer cells to sorafenib (54). These findings suggest that IL-6/STAT3 signaling pathway is a crucial factor in the occurrence of LCSCs and drug resistance.

#### The Role of IL-6/STAT3 Signaling in Promoting Malignant Transformation of Hepatocytes After HBV or HCV Infection

Chronic hepatitis is an important risk factor for stimulating the occurrence of liver cancer, including HBV and HCV. Hepatitis B virus X protein (HBx) promotes LCSCs production (55), and HCV also induces the occurrence of LCSCs (56, 57). Both HBV and HCV can promote IL-6 production and secretion in inflammation -associated cells. When acute aggravation occurs in patients with chronic hepatitis, it is also accompanied by a sharp increase concentration of IL-6, and high levels of IL-6 can further activate inflammation or tumor-related signaling pathways, thus realizing the trilogy of chronic hepatitis to cirrhosis to liver cancer. Studies have found that HBV can be involved in the translation and nuclear translocation of angiogenin (ANG) through IL-6-mediated pathways, thereby promoting tumor cell proliferation (58). Quetier et al. (59) established in a study of post hepatectomy (PH) monitoring of liver regeneration in transgenic mouse models, the results indicated that HBx expression was controlled by viral regulatory elements. The upregulation of IL-6 promotes elevated STAT3 phosphorylation levels in the liver of HBx protein transgenic mice, HBx affects the regeneration capacity of hepatocyte after PH, and HBx may be involved in accelerating cell cycle and progression of liver disease. A study of HBV-induced liver fibrosis, cirrhosis and HCC in mouse model (60), the DTNA/STAT3 signaling pathway can be activated and in turn further activates the STAT3 signaling pathway, stimulating expression of TGF-1, thus promoting the progression of HBV-induced liver fibrosis, cirrhosis, and HCC.

#### Effects of IL-6/STAT3 Signaling Pathway on the Expression of p53 and AFP in HCC Cells

P53 is one of tumor suppressor genes that most widely studied in human cancer, and the activation of p53 mainly leads to the inhibition of cancer cells growth and promotes DNA repair and apoptosis, the role is mainly mediated by its transcriptional activity. In tumor cells, accompanied by the activation of the IL-6/STAT3 signaling pathway, phosphorylated STAT3 can bind to the promoter of the *p53* gene to inhibit its transcription, thereby blocking the inhibitory effect of p53 on oncogene transcription (61). Alpha-fetoprotein (AFP) is a single-stranded serum glycoprotein, an important biomarker commonly used in the clinical diagnosis of HCC, it is a specific protein with high expression during the occurrence of liver cancer. Recent studies have found that AFP has many biological functions to promote hepatocarcinogenesis; it also plays a pivotal role in stimulating the proliferation, invasion and metastasis of HCC cells, and inhibiting HCC cells apoptosis and autophagy, and participating in immunosuppression (62–66).

Studies have shown that p53 has a repressor effect on the *afp* gene promoter (67). In HBV-related HCCs, HBx can, by interacting with p53, stimulate the expression of AFP by blocking the inhibitory effect of p53 on the promoter of *afp* gene (67). These mechanisms may be associated with the promotion of IL-6 secretion and the activation of the IL-6/STAT3 signaling pathway in HCC cells. Additionally, HBx may destroy the p53 interaction with protein partners, thereby affecting the transcriptional regulatory function of p53 and thus promoting the expression of AFP. Because AFP has an important role in promoting normal liver cell transfer to LCSCs, the IL-6/STAT3 signaling pathway may lead to the development of HCC by promoting the expression of AFP.

### Effect of the IL-6/STAT3 Signaling Pathway on the Microenvironment in HCC

The tumor microenvironment was first formally proposed in 1979, and the microenvironment is a pivotal influence factor when treating cancer (68, 69). The internal environment where the tumor is located, consists of tumor cells themselves, interstitial cells, microvessels, microlymphocytes, tissue fluid, numerous cytokines and a small number of infiltrating cells (70, 71). Hyperactivation of STAT3 is important in the microenvironmental formation of inflammatory tumors and promotes tumor proliferation and metastasis (72). The tumor microenvironment changes dramatically when chronic inflammation and fibrosis occur in liver tissue, and activation of STAT3 can induce the expression and release of cytokines, chemokines and other media associated with chronic inflammation that play a key role in inducing and maintaining the cancer-promoting inflammatory environment. Studies have found that the phagocytosis of macrophages on apoptotic bodies promotes liver fibrosis, thus accelerating the circulation of hepatocyte death and compensatory hyperplasia and eventually leading to the occurrence of HCC. Tumor-associated macrophages (TAMs) promote tumor progression by secreting IL-6 to activate IL-6/STAT3 signals in adjacent HCC stem cells in liver tissue microenvironments (52). Zheng, et al. (73) found that activation of the HCC cells IL-6/STAT3 signaling pathway was possible by upregulating the expression of tissue inhibitor of metalloproteinases-1 (TIMP-1) to stimulate the transformation of normal liver fibroblasts (LFs) toward carcinoma-associated fibroblasts (CAFs), thus promoting the initiation of liver cancer.

### Anti-Apoptotic Effect of the IL-6/STAT3 Signaling Pathway on HCC Cells

Apoptosis of HCC cells is mainly achieved by upregulating the expression of anti-apoptotic factors or promoting survival signals. After IL-6-mediated STAT3 activation, promotes the expression of anti-apoptotic protein (Bcl-xL, Bcl-2, survivin and P53, etc.) plays an important role in the anti-apoptosis of HCC cells (73–76). Bcl-2 is particularly important proteins that promotes tumor cell survival. The key factor in apoptosis due to the balance between pro- and anti-apoptotic proteins. Activation of the IL-6/STAT3 signaling pathway may increase the ratio of apoptotic factors to anti-apoptotic factors, and increased IL-6 most likely changes this ratio (77). Meanwhile, phosphorylation of STAT3 can bind directly to the promoter of the survivin gene, upregulate survivin expression and promote the survival of tumor cells; by inhibiting STAT3 activity, survivin gene expression can be downregulated to promote apoptosis of liver cancer cells (78). These findings demonstrate that activation of the IL-6/STAT3 signaling pathway can promote the expression of survival-related proteins that inhibit apoptosis of HCC cells.

### The IL-6/STAT3 Signaling Pathway Promotes Angiogenesis in Liver Cancer Tissues

Vascular endothelial growth factor (VEGF) also plays an important role in tumor invasion and metastasis, which promotes vascular endothelial cell growth and tumor neoangiogenesis. VEGF is higher expression in liver tumor tissue than in cirrhosis and normal liver tissue (79). Hypoxia is an important factor in regulating the expression of VEGF, which can induce the secretion and expression of VEGF in tumor tissue through hypoxia inducible factor 1 (HIF-1). IL-6 binds with IL-6R to induce the activation of STAT3, and activated STAT3 binds to the promoter region of the *VEGF* gene to increase transcription, promoting the formation of tumor angiogenesis. The expression of VEGF can also be promoted through HIF-1α to stimulate angiogenesis in tumor tissues. In addition, there may be a positive feedback mechanism during malignant cell transformation between STAT3 and VEGF, namely, STAT3 upregulates VEGF, while VEGF combines with the cellular surface VEGF receptor (VEGFR) to activate the IL-6/STAT3 signaling pathway to further upregulate expression of VEGF and promote the generation of blood vessels in tumor tissues. Studies have shown that STAT3 can inhibit the degradation and increase synthesis of HIF-1α. Therefore, STAT3 is necessary for endothelial cell proliferation, migration, and angiogenesis. Blocking the IL-6/STAT3 signaling pathway can inhibit endothelial cell metastasis and angiogenesis, and hinder the tumorigenesis pathway (80). Additionally, a methylation study of liver cancer indicated that low expression of IL-6 can reduce angiogenesis in HCC tissues (81), suggesting that the IL-6/STAT3 signaling pathway can promote angiogenesis in tumor tissues and provide nutrient guarantees for the development of tumorigenesis.

### Effect of the IL-6/STAT3 Signaling Pathway On The Proliferation, Invasion, and Metastasis of HCC Cells

IL-6 promotes the abnormal proliferation of cancer cells through the activation of the IL-6/STAT3 pathway, and the proliferation genes of cancer cells, such as *Ras*, *Src*, and cyclin D1, are the direct targets of STAT3 (82). Studies have found that IL-6-induced TAMs promote the amplification of CD44+ T cells to increase sphere and heterograft formation in culture, and blocking the IL-6/STAT3 signaling pathway can reduce the sphere formation ability of CD44+ T cells in the culture and growth of mouse xenotransplantation tumors (53). STAT3 inhibitors can inhibit the proliferation and development of cancers by blocking IL-6/STAT3 signaling, suppressing cancer cells proliferation and promoting apoptosis (83).

Generally, the destruction of the basement membrane is an important characteristic during cancer cells invasion. Cancer cells must penetrate the basement membrane and the natural tissue barrier formed by the extracellular matrix to undergo invasion and metastasis. Degradation of the basement membrane and the extracellular matrix is a crucial step in the invasion and metastasis of cancer cells. As the extracellular matrix degrades, cancer cells begin to infiltrate normal tissues and metastasize, and the process relies on matrix metalloproteinases (MMPs), particularly MMP-2 and MMP-9 (84, 85). MMPs interact with activator protein-1 (AP-1), of which expression is mainly regulated by the IL-6/JAK/STAT3 signaling pathway. Overexpression of MMP-9 and MMP-2 is associated with postoperative tumors in patients with liver cancer (84) and accelerates the invasion and migration capacity of HCC cells by regulating the JAK/STAT3 signaling pathway (86).

In addition, epithelial-mesenchymal transition (EMT) is closely related to primary lesion invasion and distant metastasis of cancer cells (87). The formation of EMT is mainly due to loss of the characteristics of epithelial cells, which is an important manifestation for obtaining the migration and invasion abilities of HCC cells. STAT3 is an important transcription factor in the occurrence of EMT, and studies have shown that STAT3 may play an important role in stimulating EMT through the regulation of many downstream genes (such as Snail and Twist) (88). Activated STAT3 signals are associated with Twist and calcium adhesion protein E (E-cadherin) expression and mediate the invasion and metastasis of HCC cells, and an abnormal pSTAT3/Twist/E-cadherin signal axis leads to poor prognosis in patients with liver cancer (89).

### Effect of the IL-6/STAT3 Signaling Pathway on Immune Escape of HCC Cells

The IL-6/STAT3 signaling pathway is closely related to the immune escape of HCC cells. Studies have shown that IL-6 inhibits the antigenic presentation capability of dendritic cells (DCs) by activating IL-6/STAT3 signaling pathway. IL-6 blocked the antitumor immunity reaction in tumor cells (90, 91). STAT3 activation in DCs significantly reduces tumor immune surveillance. In the tumor-bearing host, STAT3 activation from tumor cells or from normal immune cells can both inhibit the secretion of inflammatory factors and reduce the immune surveillance of tumor cells. One study demonstrated that IL-6 secretion could upregulate programmed cell death-ligand 1 (PD-L1) expression in neutrophils, thus inhibiting the activity of T cells and ultimately accelerating the immune escape of tumor cells (92). Liu et al. (93) argued that IL-6 could promote the development of liver cancer by recruiting immunosuppressive cells and excluding CD8+ T cells in tumor microenvironments, and that IL-6 may also damage the function of infiltrated T cells in tumor tissues, thus inhibiting antitumor immunity.

### Association Between the IL-6/STAT3 Signaling Pathway and Multidrug Resistance in HCC Cells

Tumor cells multidrug resistance (MDR) is a notable reason for the clinical treatment failure of liver cancer. The occurrence of MDR is an extremely complex process involving multiple factors, genes and mechanisms. Most antitumor therapies can induce inflammation by killing tumor cells and normal tissues, and in this process, the expression levels of multiple inflammatory cytokines, including IL-6, IL-8, TNF-α, and other inflammatory factors, are upregulated (94). IL-6/STAT3 is closely related to tumor cells drug resistance, and the upregulation of the tumor inflammatory factor IL-6 can promote mitogen activation of protein kinase (MAPK) through the activation of signaling pathways, such as the IL-6/STAT3, PI3K/Akt, and Ras-MAPK pathways, thus upregulating the expression of various drug-resistant proteins, such as MRP, P-gp and BCRP, and leading to HCC cells resisting drug therapy (95). Relevant studies showed that knockout of IL-6 was able to stimulate expression of E-cadherin in HCC cells, and increased the HCC cells sensitivity to sorafenib (79). These found verified that the activation of IL-6/STAT3 signaling pathway has a capacity of promotion HCC cells resist chemotherapy.

## Targeting the IL-6/STAT3 Signaling Pathway Can Improve the Clinical Treatment of Liver Cancer

At present, tumor-targeted therapy has great promising prospect, and the current targeted IL-6/STAT3 signaling pathway is a main biological therapy concentrated on IL-6 blocking antibodies, IL-6 receptor blocking antibodies and specific STAT3 inhibitors. Experiments concentrated on multiple osteomyelitis, rheumatoid arthritis/malignant tumors, etc., also shows a certain curative effect. The results of multiple preclinical trials showed significant inhibition of tumor growth, both alone and combined with chemotherapy. The IL-6/STAT3 signaling pathway is closely related to HCC initiation, development, metastasis, and drug resistance, which is continuously activated and overexpressed in a variety of tumor cells and has become a hot spot in cancer treatment. This pathway has positive significance for the treatment of HCC and others cancers by blocking the IL-6/STAT3 signaling pathway.

### Anti-IL-6 and IL-6R Antibodies

There is increasing evidence that IL-6 is a therapeutic target for several cancers, reducing phagocytosis and migration mediated by STAT3 phosphorylation and by neutralizing IL-6 or antagonistic IL-6R in cancer cells. Studies have shown that apoptosis of HCC cells is facilitated by interfering the role and expression of IL-6, suggesting that blocking IL-6 can be used as a potential treatment for the sorafenib sensitivity of HCC cells (96). Evidence has also verified that anti-PD-L1 resistance can be reversed by blocking IL-6, which provides a potential strategy for overcoming the resistance of anti-PD-L1 in liver cancer (93). Recently, studies indicated that Madindoline A, a small molecule for inhibiting the dimerization of IL-6/IL-6R/gpl30 trimeric complexes, inhibits the growth of HCC cell line, HepG2 cells (97). Siltuximab and CNTO-136 are able to neutralize the activity of IL-6 (98, 99); Suppressing the activity of IL-6 by ALD518, and blocking IL-6R by monoclonal antibody(Tocilizumab) is an effective strategy for treatment of some cancers, such as non-small cell lung cancer (100), multiple myeloma (101), epithelial ovarian cancer (102), B-cell lymphoma (103), renal cell carcinoma (104) etc. These studies imply that blocking IL-6R is applied to therapeutic of HCC.

### JAK Inhibitors

Numerous clinical studies have shown that JAK-specific inhibitors can reduce growth in various tumor models *in vivo*, including liver, pancreatic, brain, colorectal, stomach, lung, ovarian, and breast cancer. The studies revealed the effect of the JAK inhibitor Ruxolitinib on HCC cells, the results showed that ruxolitinib could effectively inhibit the JAK/STAT signaling pathway in HCC cells and significantly reduce the expression of the downstream JAK target pSTAT3 (105). Ruxolitinib also significantly reduces the proliferation and colony formation of HCC cells (105). AG490 is an artificial ester derivative of phenylacrylonitrile that effectively blocks JAK activation by competitively binding tyrosine kinase binding sites and thus suppresses STAT3 activation. By studying AG490, Thompson et al. (106) used it in *in vitro* experiments and found that the inhibitor AG490 significantly reduced the vitality of hepatoma SMMC-7721 cells, thus inhibiting the growth of xenotransplanted HCC cells. AZG1480 and TG101209 inhibit the activity of JAK to suppress the growth of HCC (106, 107). These results prove that JAK inhibitors are able to inhibit activation of STAT3 to suppress the growth of HCC cells.

### STAT3 Inhibitors

**Figure 5:**
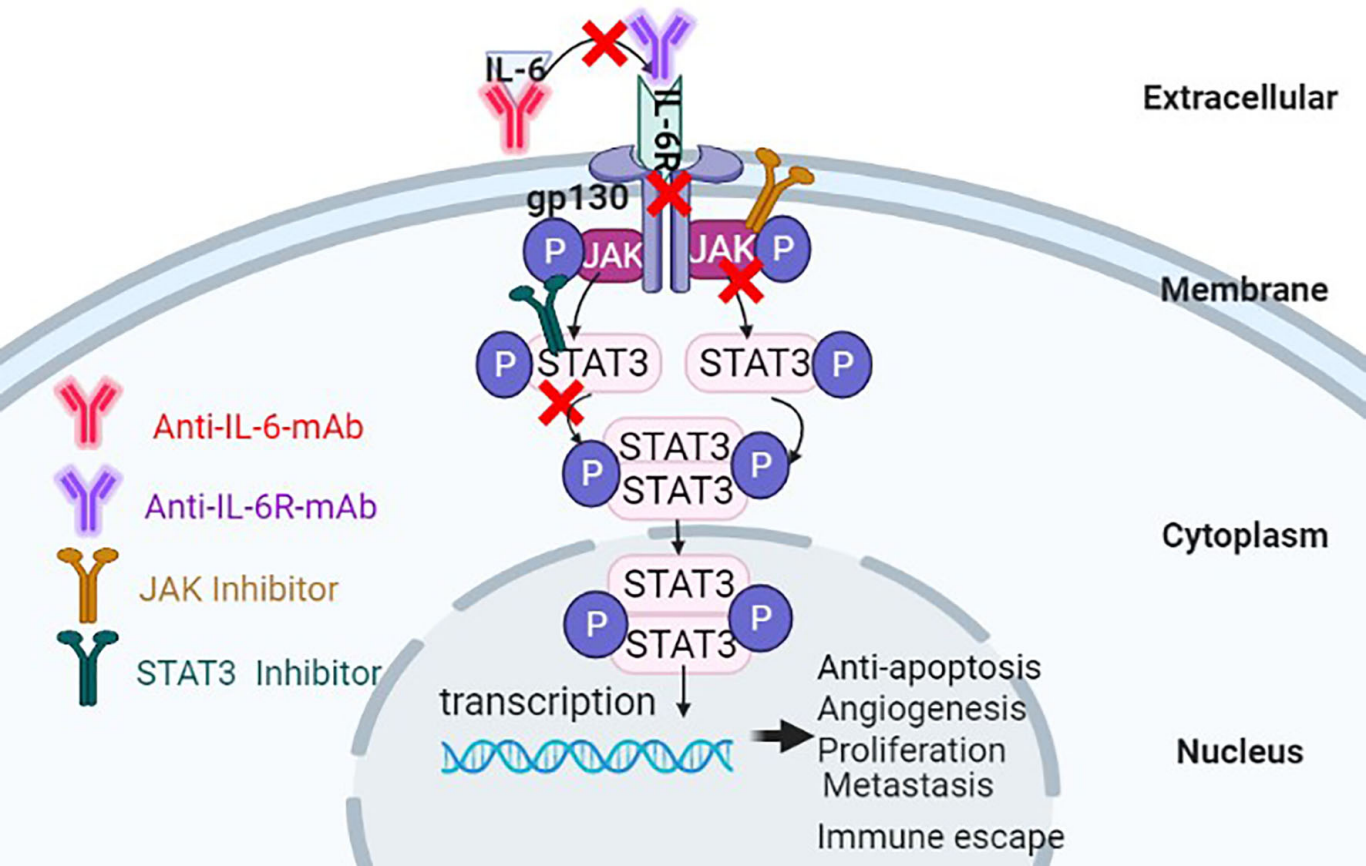
Antitumor effects of suppressing the IL-6/STAT3 signaling pathway through multiple approaches.

STAT3 inhibitors can be classified into small molecular types, oligonucleotide types, peptide analogs, natural product derivatives, etc., according to the structure, the inhibitors can be divided into targeted SH2 domain inhibitors, targeted DNA binding domain inhibitors, targeted N-terminal domain inhibitors, STAT3 oligonucleotide inhibitors, etc. STAT3 region targeting inhibitors can suppress the proliferation, survival, and differentiation of HCC cells by preventing phosphorylation of STAT3, inhibiting the formation of STAT3 dimers, or interfering with their activity of interaction with DNA sequences. Jung et al. (108) showed that the proliferation of HCC cell was blocked by the small molecule STAT3 inhibitor C188-9. Gene expression analysis showed that C188-9-treated HepPten(-) mice had inhibited signaling pathways downstream of STAT3. The STAT3 small-molecule inhibitor LLL12 plays a role in blocking IL-6-induced STAT3 phosphorylation and nuclear translocation, thus inhibiting proliferation and promoting apoptosis of HCC cells (109), C188-9, Curcumin, OPB-31121, S31-201 and AZD9150 inhibit the phosphorylation of STAT3 to block the proliferation of HCC cells (108, 110–113). Studies also indicated that destroyed the structure of STAT3 dimer or inhibited the dimerization of STAT3 by small molecule, S3I-1757, STA-21 also can regress human cancer cells in xenografts animal model and abnormal proliferation disorders (114, 115). These evidences implicate that STAT3 inhibitors play important role in blocking the activation of IL-6/STAT3 signaling pathway, lead to inhibit the proliferation of HCC cells. The effect on inhibiting IL-6/STAT3 signal pathway for treatment of cancer is displayed in below table (see **Table 1** and **Figure 5**).

**Table 1:** Multiple treatments targeting the IL-6, IL-6R and IL-6-related signaling pathways.

Target	Inhibitor/drug	Effect	References
IL-6/IL-6R	Madindoline A	Inhibits the dimerization of IL-6/IL-6R/gpl30 trimeric complexes	(97)
	Siltuximab	Neutralize the activity of IL-6	(98)
	Humanized anti-IL-6 antibody (ALD518)	Neutralize the activity of IL-6	(100, 101)
	CNTO-136	Neutralize the activity of IL-6	(99)
	Tocilizumab	inhibits the binding of IL-6 to IL-6R	(102)
JAK	AG490	Inhibit the activity of JAK	(106)
	Ruxolitinib	Inhibit the activity of JAK	(105)
	AZG1480, ZAD9150	Inhibit the activity of JAK	(110, 111)
	TG101209	Inhibit the activity of JAK	(106)
STAT3	S31-201	inhibits the activity of STAT3	(110)
	C188-9	Inhibit the phosphorylation of STAT3	(111)
	Curcumin	Inhibit the phosphorylation of STAT3	(112)
	LLL12	Inhibit phosphorylation and nuclear translocation of STAT3	(109)
	OPB-31121	Inhibit the phosphorylation of Tyr705	(111)
	AZD9150	Inhibit the phosphorylation of STAT3	(112)
	S3I-1757	Destroy the structure of STAT3 dimer by binding to site Tyr705	(114)
	STA-21	Inhibit the dimerization of STAT3 and binding to DNA	(115)

## Future and Outlook

Molecular targeted-therapy is an advanced scientific technology in the clinical treatment of cancer, but none of many molecular targeted drugs are completely designed for liver cancer. It is difficult to develop targeted drugs to treat liver cancer. Exploration of its cause is as follows: (1) The occurrence of liver cancer involves multiple factors and the complex, the developmental mechanism is still unclear, and specific well-directed development of targeted drugs is difficult. Simultaneously, normal liver cells possess their own characteristics and rapid proliferation; once tumorigenesis, the difference is arise in the proliferation and heterogeneity of HCC cells, and it is not easy to find specific treatment targets. (2) At present, most targeted drugs treatment emerge low efficiency and poor efficacy. (3) Targeted drugs are not highly selective for targeting HCC cells, and there are toxicity and side effects with “off-target effects” and high drug resistance. Expensive costs of research and development can be prohibitive for wide use. (4) Patients with liver cancer may have different responses to targeted drug therapy, with differences in race and sex, and there is still a lack of effective methods to detect molecular changes in HCC cells. With the development of many advanced biotechnologies and exploration of the genesis mechanism of HCC, the treatment of liver cancer is facing new opportunities and challenges. Molecular targeted therapy will gradually become the favored method and represents the development direction of liver cancer treatment in the future. The relationship between IL-6/STAT3 signaling pathway characteristics and their mediated physiological function needs to be further interpreted. Meanwhile, inhibitors of the IL-6/STAT3 signaling pathway should be promoted, and the efficacy and safety of these targeted inhibitors should be evaluated, it is need to formulate the standardization of clinical individualization treatment for liver cancer.

The occurrence of liver cancer is closely associated with inflammation. IL-6 is an important member of the inflammatory cytokine network. In recent years, an increasing number of studies have revealed that the IL-6/STAT3 signaling pathway plays a pivotal role in the development of liver cancer, and many studies have shown that inhibition of the IL-6/STAT3 signaling pathway can block the occurrence and progress of HCC. This signaling pathway is still a hot spot of research for cancer treatment. IL-6/STAT3 is a pivotal signaling pathway to promote expression of PD-L1 in HCC cells (116), leading to escape immune surveillance of HCC cells. Meanwhile, tumor infiltrating immune cells secreted IL-6 is able to stimulate IL-6/STAT3 signaling pathway to promote the malignant behaviors of HCC cells. In future, blocking the secretion of IL-6 and synergizing with the inhibitors of IL-6/STAT3 pathway signaling is a more effective application prospect for targeting therapeutic of HCC (117–119) Also, as previously mentioned, the IL-6/STAT3 signaling pathway may lead to the development of HCC by promoting the expression of AFP. AFP is specifically expressed in liver cancer patients. AFP is a very complex biological activity protein whose biological function is not fully clear and needs further research. The study of the IL-6/STAT3 signaling pathway in clinical trials of HCC is still limited, because the expression of AFP is activated by the IL-6/STAT3 signaling pathway. Inhibits the expression and role of AFP, which may be a promising strategy for blocking IL-6/STAT3 to stimulate drug resistance, proliferation, invasion, metastasis and recurrence of HCC. More studies are expected to demonstrate that additional new drugs can have a role in blocking this signaling pathway in the future, these project is able to bring new breakthroughs to the clinical treatment of patients loading HCC.

## Author Contributions

JX, HL, GW, and MZ gathered the related literature, prepared the figures and drafted the manuscript. MZ and ML participated in the design of the review and drafted the manuscript. All authors contributed to the article and approved the submitted version.

## Funding

This work is supported by the Natural Science Foundation of Hainan Province (Nos: 820QN403, 2019CXTD406, 2019CR204 and 20168263), Hainan Health Industry Scientific Research Project Foundation (No. 20A200496), the National Natural Science Foundation of China (Nos.82060514, 81960519, 81660463); and the Hainan Provincial Association for Science and Technology Program of Youth Science Talent and Academic Innovation (No. QCXM 201922), Research Project of Take off the Proclamation and Leadership in Hainan Medical College Natural Science Foundation (No. JBGS202106).

## Conflict of Interest

The authors declare that the research was conducted in the absence of any commercial or financial relationships that could be construed as a potential conflict of interest.

## Publisher’s Note

All claims expressed in this article are solely those of the authors and do not necessarily represent those of their affiliated organizations, or those of the publisher, the editors and the reviewers. Any product that may be evaluated in this article, or claim that may be made by its manufacturer, is not guaranteed or endorsed by the publisher.

## Abbreviations

AFP, Alpha-fetoprotein; ANG, Angiogenin; AP-1,Activator protein-1; APRF, Acute phase response factor; CCD, Coiled-coil domain; CAFs, Carcinoma-associated fibroblasts; DC, Dendritic cell; DBD,DNA binding domain; EGFR, Epidermal growth factor receptor; EMT, epithelial-mesenchymal transition; HCC, hepatocellular carcinoma; HBV, hepatitis B virus; HBx, Hepatitis B virus X protein; HCV, hepatitis C virus; HIF-1,Hypoxia inducible factor 1; IFN, Interferon; IL-1β,Interleukin-1β; IL-6,interleukin-6; IL-6R,interleukin-6 receptor; LD, Linker domain; LCSCs, liver cancer stem cells; MAPK, Mitogen Activated Protein Kinase; mIL-6R, membrane- bound IL-6R; MAPK, Mitogen-activated protein kinase; MDR, multidrug resistance; MMP, Matrix metalloproteinase; PD-L1,Programmed cell death-ligand 1; PH, pleckstrin homology; PI3K, phosphatidylinositol 3-kinase; PIAS, Protein inhibitors of activated stats; PTP, Protein tyrosine phosphatases; RTK, Receptor Tyrosine Kinases; SH2,Src homology 2; SOCS, Suppressors of cytokine signaling; STAT3,Signal transducer and activator of transcription 3;STATs,signal transducer and activator of transcription proteins; TAMs, tumor-associated macrophages; TIMP-1,Tissue Inhibitor of Metalloproteinases-1; TNF, Tumor necrosis factor; VEGF, Vascular endothelial growth factor.
